# Supplementary material for: Resolving the full spectrum of human genome variation using Linked-Reads
Source: Genome Res. 2019 Apr;29(4):635–45. doi: 10.1101/gr.234443.118 (PMC6442396; doi:10.1101/gr.234443.118)
Supplement: Supplemental Material [file supp_gr.234443.118_Supplemental_longranger-2.2.zip › longranger-2.2.tar.bz2!/lr2.2-public/lib/stan/cmdstan/stan/doc/api/html/functions_func_l.html]

Stan: Class Members - Functions


|  |  |
| --- | --- |
|  | Stan  2.5.0  probability, sampling & optimization |


- Main Page
- Related Pages
- Namespaces
- Classes
- Files

- Class List
- Class Index
- Class Hierarchy
- Class Members

- All
- Functions
- Variables
- Typedefs
- Enumerator
- Related Functions

- a
- b
- c
- d
- e
- f
- g
- h
- i
- j
- k
- l
- m
- n
- o
- p
- q
- r
- s
- t
- u
- v
- w
- x
- z
- ~


All Classes Namespaces Files Functions Variables Typedefs Enumerations Enumerator Friends Macros Pages

### - l -

- LBFGSUpdate()
  : stan::optimization::LBFGSUpdate< Scalar, DimAtCompile >
- LDLT\_alloc()
  : stan::agrad::LDLT\_alloc< R, C >
- LDLT\_factor()
  : stan::math::LDLT\_factor< stan::agrad::var, R, C >
  , stan::math::LDLT\_factor< T, R, C >
- learn\_covariance()
  : stan::mcmc::covar\_adaptation
- learn\_stepsize()
  : stan::mcmc::stepsize\_adaptation
- learn\_variance()
  : stan::mcmc::var\_adaptation
- list\_argument()
  : stan::gm::list\_argument
- local\_var\_decl\_visgen()
  : stan::gm::local\_var\_decl\_visgen
- log\_abs\_det()
  : stan::agrad::LDLT\_alloc< R, C >
  , stan::math::LDLT\_factor< T, R, C >
- log\_prob()
  : stan::mcmc::sample
- logp()
  : stan::optimization::BFGSLineSearch< M, QNUpdateType, Scalar, DimAtCompile >
- lowest()
  : Eigen::NumTraits< stan::agrad::var >
- LSOptions()
  : stan::optimization::LSOptions< Scalar >


---

[ Stan Home Page ]

*© 2011–2014,
Stan Development Team.*
